# Tissue-Specific Factors Differentially Regulate the Expression of Antigen-Processing Enzymes During Dendritic Cell Ontogeny

**DOI:** 10.3389/fimmu.2020.00453

**Published:** 2020-03-31

**Authors:** Karim Mahiddine, Chervin Hassel, Claire Murat, Maeva Girard, Sylvie Guerder

**Affiliations:** Centre de Physiopathologie de Toulouse Purpan, Institut National de la Santé et de la Recherche Médicale, Centre National de la Recherche Scientifique, Université Paul Sabatier Toulouse III, Toulouse, France

**Keywords:** dendritic cell ontogeny, thymus-specific serine protease, cathepsin S, cathepsin L, thymic dendritic cell, splenic dendritic cell

## Abstract

Dendritic cells (DCs) form a collection of antigen-presenting cells (APCs) that are distributed throughout the body. Conventional DCs (cDCs), which include the cDC1 and cDC2 subsets, and plasmacytoid DCs (pDCs) constitute the two major ontogenically distinct DC populations. The pDCs complete their differentiation in the bone marrow (BM), whereas the cDC subsets derive from pre-committed BM precursors, the pre-cDC, that seed lymphoid and non-lymphoid tissues where they further differentiate into mature cDC1 and cDC2. Within different tissues, cDCs express distinct phenotype and function. Notably, cDCs in the thymus are exquisitely efficient at processing and presenting antigens in the class II pathway, whereas in the spleen they do so only upon maturation induced by danger signals. To appraise this functional heterogeneity, we examined the regulation of the expression of distinct antigen-processing enzymes during DC ontogeny. We analyzed the expression of cathepsin S (CTSS), cathepsin L (CTSL), and thymus-specific serine protease (TSSP), three major antigen-processing enzymes regulating class II presentation in cDC, by DC BM precursors and immature and mature cDCs from the spleen and thymus. We found that pre-cDCs in the BM express relatively high levels of these different proteases. Then, their expression is modulated in a tissue-specific and subset-specific manner with immature and mature thymic cDCs expressing overall higher levels than immature splenic cDCs. On the other hand, the TSSP expression level is selectively down-regulated in spleen pDCs, whereas CTSS and CTSL are both increased in thymic and splenic pDCs. Hence, tissue-specific factors program the expression levels of these different proteases during DC differentiation, thus conferring tissue-specific function to the different DC subsets.

## Introduction

Dendritic cells (DCs) are specialized antigen-presenting cells (APCs) that are essential for effective immunity and tolerance. Distinct subsets of DCs are distributed throughout the body and can be distinguished based on phenotypic markers, transcriptional programs, tissue distribution, and function (1, 2).

Currently, DCs are subdivided into two major ontogenically distinct populations, the conventional DCs (cDCs) and the plasmacytoid DCs (pDCs). The CD11c^high^ CMHII^high^ cDC population is further subdivided into two subsets presenting distinct phenotype and function, the cDC1 (CD24^+^ XCR1^+^ CD11b^low^ Sirpα^low^) and cDC2 (CD24^low^ XCR1^−^ CD11b^+^ Sirpα^+^) subsets (3). Both cDC subsets are specialized antigen-processing cells and APCs and as such are the most efficient DC subset at priming and polarizing T cells (1). However, the cDC1 subset is exquisitely efficient at cross-presenting exogenous antigen in the class I pathway. pDCs, on the other hand, produce copious amount of type I interferon (IFN) and inflammatory cytokines in responses to danger signals (4).

The distinct DC subsets arise from a common DC progenitor (CDP). Within the bone marrow (BM), the CDP generates both pDC and the precursor of cDC (pre-cDC), which in turn commits to pre-cDC1 and pre-cDC2 progenitors (5–9). Committed pre-cDC1 and pre-cDC2 precursors seed lymphoid and non-lymphoid tissue and further differentiate into immature cDC1 and cDC2, respectively. In contrast, pDCs complete their differentiation within the BM and then migrate through the bloodstream to lymphoid organs (4).

Several studies showed that cDCs isolated from different lymphoid and non-lymphoid tissues express distinct phenotype and function, which correlates with the expression of tissue-specific transcriptional programs (2). Notably, thymic cDCs have a more mature phenotype than splenic cDCs, expressing higher levels of MHC class II, CD86, and CD40 (10–12). Furthermore, thymic cDCs efficiently present exogenous antigens to both CD4 and CD8 T cells, whereas spleen cDCs do so only following toll-like receptor (TLR) stimulation (10, 13). This is at least in part due to the homeostatic maturation of thymic cDCs, with mature thymic cDCs being exquisitely efficient at inducing central tolerance (12).

The presentation of exogenous proteins in the class II pathway relies on sequential proteolysis of endocytosed proteins by endosomal proteases (14). DCs express several antigen-processing enzymes of the cathepsin family of cysteine and aspartyl proteases including the cathepsin S (CTSS) and cathepsin L (CTSL) protease (15). In addition, we showed that thymic cDCs uniquely express the thymus-specific serine protease (TSSP). In thymic cDCs, TSSP limits the presentation of several self-antigens and thus limits the deletion of self-reactive CD4 T cells (16–19). Consequently, TSSP-deficient non-obese diabetic (NOD) mice are completely protected from autoimmune diabetes and develop less severe experimental autoimmune encephalomyelitis (16, 20).

Although the role of these proteases has been widely studied during central tolerance or induction of immune responses *in vivo*, their expression pattern during DC ontogeny or by the cDC subsets of different tissues is still poorly characterized. Here, we analyzed the expression of TSSP, CTSS, and CTSL expression by DC BM precursors and immature cDCs and pDCs in the spleen and thymus. Collectively, our results show that the expression of these different proteases is determined during DC differentiation in a tissue-specific and subset-specific manner. Hence, tissue-specific factors imprint the function to the different DC subsets during their differentiation in the spleen and thymus.

## Materials and Methods

### Mice and Treatment

NOD/LtJ (NOD) mice were maintained at the UMS006 animal facility (Toulouse). C57Bl6/JRj (B6) mice were purchased from Janvier Labs (Le Genest Saint Isle, France).

For the *in vivo* activation of DC subsets, mice were i.v. injected with 5 μg of lipopolysaccharide (LPS) from *Escherichia coli* 0111:B4), 10 μg of CpG-B ODN1826, and 10 μg of Poly I:C (InvivoGen, Toulouse, France).

For *in vivo* FLT3L treatment, mice were injected subcutaneously (s.c.) with 5 × 10^6^ B16-Flt3L melanoma cells (21).

All experiments involving animals were performed in accordance with national and European regulations and the Institut National de la Santé et de la Recherche Médicale institutional guidelines. Protocols were approved by the “Midi Pyrénées” ethical committee.

### Isolation of Splenic Dendritic Cells, Thymic Dendritic Cells, and Bone Marrow Precursor of Conventional Dendritic Cells and Plasmacytoid Dendritic Cells

For *ex vivo* DC isolation, the thymuses or spleens were pooled and cut with blunt scissors before digestion in Roswell Park Memorial Institute (RPMI) 1640 with 2% fetal bovine serum (FCS) supplemented with Liberase (200 μg/ml, Roche) and DNase I (40 μg/ml, Sigma-Aldrich) for 10–15 min. After incubation with 2.4G2 antibody, DCs were isolated using CD11c MicroBeads (Miltenyi Biotec), according to manufacturer's instruction. Each of the cDC subsets and pDCs was subsequently examined by fluorescence-activated cell sorting using a FACSAria™ II high-speed cell sorter (BD Biosciences) following staining with F4-80-, CD45.1- or CD45.2-, CD11c-, CD11b-, B220-, CD24-, and Sirpα-specific antibodies.

For pre-cDC and pDC isolation, BM was extracted from the femurs and tibias, and erythrocytes removed using Gey's treatment. Lineage cells were first removed using fluorescein isothiocyanate (FITC) conjugate anti-CD3, anti-CD19, anti-NKP46, and, for pre-cDC isolation only, anti-B220, and anti-FITC magnetic MicroBeads (Miltenyi Biotec). Pre-cDCs and pDCs were subsequently FACS-sorted following staining with anti-MHC II-, CD135-, Sirpα- and CD11c-specific antibodies or CD19-, B220-, and CD11c-specific antibodies, respectively.

### *In vitro* Cell Culture

BM cells were extracted, and erythrocytes were removed using Gey's buffer. Cells were cultured for 10 days at a density of 1.5 × 10^6^ cells in a 24-well plate in complete RPMI 1640 medium with 10% FCS containing recombinant mouse 100 ng/ml FLT3L (Miltenyi Biotec), at 37°C in 5% CO_2_. Half media were exchanged at day 3 with fresh media containing 50 ng/ml of FLT3L (22).

### Antibodies and Flow Cytometry

Cells were stained with a combination of biotinylated, FITC-, PE-, PE-Cy7, allophycocyanin-, allophycocyanin-eFluor780-, allophycocyanin-Cy7-, Brilliant Violet 421-, Brilliant Violet 786-, Pacific Blue-, Brilliant Violet 605-, Pacific Orange-, PerCPVio700, or PerCP-Cy5.5-conjugated monoclonal antibodies. The anti-CD45.1 (clone A20), CD45.2 (104), F4/80 (BM8), CD11c (HL3 or N418), CD11b (M1/70), B220 (RA3-6B2), CD24 (M1/69), CD172α (P84), CD80 (16-10A1), CD86 (GL1), MHC II (OX-6 or m5/114), CD3 (1145-2C11 or 500A2), CD19 (1D3), NKP46 (29A1.4), CD135 (A2F10.1), CCR7 (4B12), XCR1 (ZET), Ly6G (1A8), CD19 (1D3), CD90.2 (53-2.1), CD64 (X54-5/7.1), CCR9 (REA943), SiglecH (eBio440c), PDCA-1 (eBio927), and ESAM (REA722) antibodies were from eBioscience, BD Biosciences, or Miltenyi Biotec. Events were collected within a lymphoid gate based on forward-scatter (FSC) and side-scatter (SSC) profiles, and dead cells were excluded using propidium iodide or Fixable Viability Dye staining. Data were acquired on a BD LSRFortessa™ flow cytometer (BD Biosciences). Data were analyzed using BD FACSDiva™ software (BD Biosciences) or FlowJo software (Tree-star).

### RNA Isolation and RT-qPCR Analysis

RNA was extracted using a RNA XS Isolation Kit (Macherey Nagel) or RNeasy Mini Kit (QIAGEN), and cDNA was synthetized using SuperScript™ II reverse transcriptase (Invitrogen) and random primers (Invitrogen) according to manufacturers' instructions. A LightCycler® 480 Real-Time PCR System (Roche) was used for quantitative PCR. Results were analyzed with LightCycler 480 V1.5 software. The cycling threshold value of the endogenous control gene (HPRT) was subtracted from the cycling threshold value of each target gene to generate the change in cycling threshold (ΔC_T_). The sequences of the primers for target genes are listed in Table 1.

**Table 1:** Sequences of the primers used for RT-qPCR.

HPRT	Sens	CTGATAAAATCTACAGTCATAGGAATGGA
	Antisens	AGCCCTCTGTGTGCTCAAGG
TSSP	Sens	CGCAGCATGGGACAGAAGTGTTTA
	Antisens	ACTGAAGACCCTCACAGGTGACAT
Cathepsin S	Sens	TCAGAACCTGGTGGACTGCTCAAA
	Antisens	TGGCTTTGTAGGGATAGGAAGCGT
Cathepsin L	Sens	TGTAGCAGCAAGAACCTCGACCAT
	Antisens	TGGTTGTCCCGGTCTTTGGCTATT

*TSSP, thymus-specific serine protease*.

### RNAseq Analysis

Immature and mature thymic and spleen cDC1 and cDC2 were FACS-sorted based on CCR7 and ESAM expression. RNA was extracted using a RNA XS Isolation Kit (Macherey Nagel), and total RNA's quality and quantity were determined using an Agilent 2100 Bioanalyzer (Agilent Technologies) and a Qubit 2.0 Fluorometer (Life Technologies). Libraries were generated using illumina® Truseq Stranded mRNA library following manufacturer's instructions. Each RNAseq library was sequenced in triplicate on Illumina HiSeq 3000 sequencer using 150 bp/sequence paired-end “reads” at Genotoul genomic platform (Castanet-Tolosan, France). Between almost 70 and 90 million “reads” were obtained per sample. “Reads” were trimmed through use of the Cutadapt tool (version 1.3), with removal of low-quality bases (–*q* value, <10) and clipping of adaptor sequences. High-quality RNAseq “reads” were aligned to the mouse reference genome mm10 (National Center for Biotechnology Information) with STAR software (version 2.6.0). The Python package HTSeq-count was used to count the number of reads overlapping with each gene using ENSEMBL annotation. Differential expression genes between cDC subpopulations were determined with DESeq2 package of Bioconductor software, with an adjusted *P*-value of <0.1 (*P*-value adjusted for multiple testing with the Benjamini–Hochberg procedure) as the cutoff for genes with significantly differential expression in one cell population relative to their expression in another cell population. Ingenuity Pathway Analysis (IPA; QIAGEN Inc., Hilden, Germany) was then used to translate the differential expressed genes into canonical pathways using the Ingenuity Knowledge Base. Two statistical indexes (*P*-value and *z*-score) are determined for each inference. The *P*-value indicates significantly enriched pathways, and the *z*-score represents the statistical measure of the concordance between differentially expressed genes (DEGs) and the associated canonical pathway.

### Statistical Analysis

Statistical analyses were performed in GraphPad Prism. *P*-values were determined using a two-tailed Mann–Whitney test except for Supplemental Figure 2 where a parametric *t*-test was used. In cases of multiple comparisons, the data were first analyzed by the Kruskal–Wallis test, and then Mann–Whitney-derived *P*-values were corrected using the Bonferroni adjustment. Statistical significance was defined as *P* < 0.05.

For RNAseq analysis significance, DESeq2 uses a Wald test *P*-value, which is adjusted for multiple testing using the procedure of Benjamini–Hochberg.

## Results

### Thymic Dendritic Cells Express Overall Higher Levels of Antigen-Processing Enzymes

**Figure 1:**
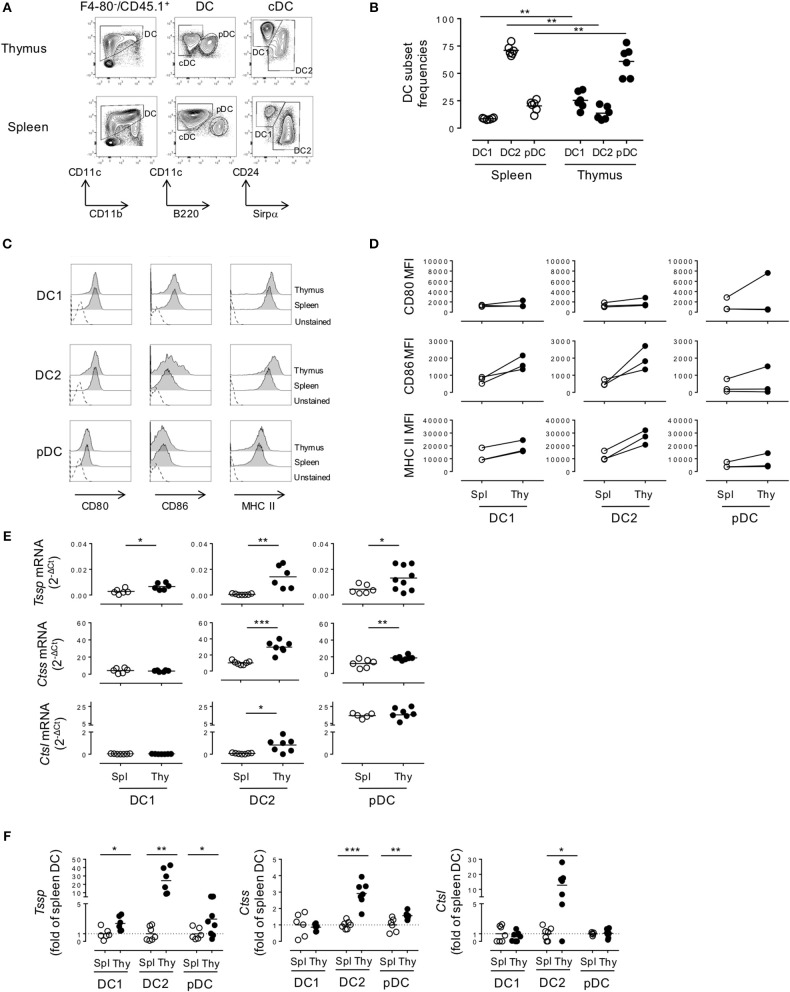
Thymic dendritic cells (DCs) express overall higher levels of antigen-processing enzymes. **(A)** Phenotypic analysis of thymic and splenic DC subsets of non-obese diabetic (NOD) mice, after selection of F4-80^−^CD45^+^ live cells. **(B)** cDC1 (CD11c^+^B220^−^CD24^+^Sirpα^−^), cDC2 (CD11c^+^B220^−^CD24^−^Sirpα^+^), and plasmacytoid DCs (pDCs) (CD11c^+^B220^+^) frequencies in the spleen or in the thymus are indicated. **(C)** Histogram overlay analysis of thymic and splenic DCs subset of NOD mice for CD80, CD86 co-stimulation markers, and MHC class II molecules. **(D)** Comparative median fluorescence intensity for CD80, CD86, and class II surface expression by splenic and thymic cDC1, cDC2, and pDCs. **(E)** Quantitative real-time RT-PCR analysis of *Tssp, Ctss*, and *Ctsl* mRNA levels in cDC1, cDC2, and pDCs isolated from thymus or spleen of NOD mice. Each symbol corresponds to a pool of 10 (thymus) or 5 (spleen) mice analyzed in 8–10 experiments. **(F)** *Tssp, Ctss*, and *Ctsl* mRNA levels in NOD mice from **(D)** is presented as fold expression normalized to the corresponding spleen DC subset. Significant *P*-values are indicated (*P < 0.05; **P < 0.01; ***P < 0.001).

As a first step to the understanding of the regulation of the expression of different antigen-processing enzymes by the different DC subsets found in spleen and thymus of NOD mice, we compared their representation and maturation in each tissue. Both cDCs and pDCs were characterized by their expression of CD11c and the absence of the macrophage-specific marker F4/80. Thus, the cDC1 and cDC2 subsets were defined as F4/80^−^CD45.1^+^CD11c^+^B220^−^CD24^+^ or F4/80^−^CD45.1^+^CD11c^+^B220^−^Sirpα^+^ cells, respectively (Figure 1A). Consistent with the use of CD24 as a specific marker to discriminate cDC1 from cDC2 subset, we found that 91 ± 1.2% and 88.2 ± 1.7% of thymic and splenic CD11c^+^B220^−^CD24^+^ cells, respectively, expressed the cDC1 conventional marker XCR1, whereas its expression was barely detectable among the CD11c^+^B220^−^CD24^−^Sirpα^+^ cDC2 subset (Supplemental Figure 1A). The pDC subset was defined as F4/80^−^CD45.1^+^CD11c^+^B220^+^ (Figure 1A). Of note, thymic and splenic CD11c^+^B220^+^ cells were positive for PDCA-1 expression, more than 80% of this population was also CCR9^+^/SiglecH^+^ positive, consistent with our strategy using CD11c^+^B220^+^ to define the bona fide pDC populations (Supplemental Figure 1B). We found that the relative representation of the different DC subsets was distinct between spleen and thymus (Figure 1B), in agreement with published data. Indeed, although the cDC2 subset was the most preponderant subset in the spleen (cDC2 = 71.1 ± 1.9% (mean ± SEM) vs. cDC1 = 8.6 ± 0.3%, *n* = 6), it was under-represented in the thymus (cDC2 = 13.7 ± 2.5% vs. cDC1 = 25.4 ± 3.2%, *n* = 6). The relative frequency of the pDC subset also diverged drastically between the spleen and thymus (2.3 ± 2.2 in the spleen vs. 61.0 ± 5.6% in the thymus). In addition, on the basis of CD86 and MHC class II expression, we found that thymic cDCs show a more mature phenotype than their splenic counterparts (Figures 1C,D).

We next determined whether these phenotypic differences between thymic and splenic DCs were also correlated with difference in the expression levels of proteases of the class II presentation pathway, namely, TSSP, CTSS, and CTSL. We therefore FACS-sorted each individual subset and analyzed the expression of the different proteases by RT-qPCR (Figures 1E,F). We found that *Tssp* was barely expressed by the different splenic DC subsets and expressed at significantly higher levels by all thymic DC subsets (Figure 1E, upper row and Figure 1F, left panel). It is worth noting that thymic cDC2 and pDCs had the highest levels of *Tssp* mRNA. By contrast, *Ctss* was expressed by all splenic and thymic DC subsets; but, as observed for *Tssp* mRNA, thymic cDC2 and pDCs expressed significantly higher levels of *Ctss* mRNA than their splenic counterpart (Figure 1E, middle row and Figure 1F, middle panel). Although *Ctsl* is highly expressed by cortical thymic epithelial cells, its expression was also extended to other APCs including macrophages and pDCs (15, 23, 24). We found that the *Ctsl* mRNA level was very low in both cDC subsets and similarly high in the pDC subset of the spleen and thymus. Moreover, a significant increase in *Ctsl* transcripts was observed in thymic cDC2 compared with their splenic counterparts (Figure 1E, lower row and Figure 1F, right panel).

Collectively, these different results showed that TSSP is selectively expressed by thymic DCs and that, overall, thymic DCs expressed higher levels of the different antigen-processing enzymes examined.

**Figure 2:**
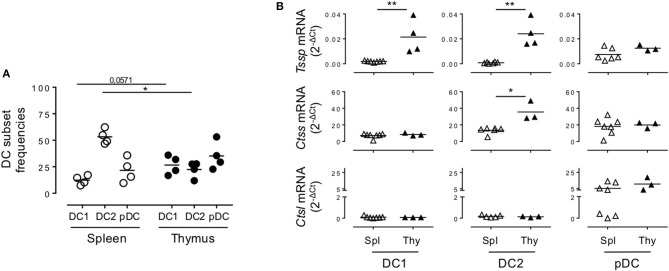
Non-obese diabetic (NOD) and B6 mice express comparable levels of *Tssp, Ctss*, and *Ctsl* mRNA. **(A)** The dendritic cell (DC) subsets of B6 mice spleen and thymus were identified as described in Figure 1A. cDC1 (CD11c^+^B220^−^CD24^+^Sirpα^−^), cDC2 (CD11c^+^B220^−^CD24^−^Sirpα^+^), and plasmacytoid DC (pDC) (CD11c^+^B220^+^) frequencies in the spleen or in the thymus of B6 mice are indicated. **(B)** cDC1, cDC2, and pDCs were FACS-sorted from the spleen and thymus of B6 mice and analyzed for *Tssp, Ctss*, and *Ctsl* expression by RT-qPCR. Each symbol corresponds to a pool of 5 (spleen) or 10 (thymus) mice analyzed in four independent experiments. Significant *P*-values are indicated (*P < 0.05; **P < 0.01).

Given that NOD mice present several genetic defects and develop autoimmune pathologies linked to defects in central tolerance, we wondered if the protease expression pattern observed in NOD mice was unique to that strain of mice or was also observed in non-autoimmune prone mouse strains. We therefore similarly cell sorted each individual subset and analyzed *Tssp, Ctss*, and *Ctsl* expression patterns in the different thymic and splenic DC subsets of B6 mice by RT-qPCR (Figure 2). Of note, we found that the frequency of the different DC subsets differs between NOD and B6 mice (compare Figure 1B with Figure 2A), consistent with previous studies highlighting the unusual DC distribution in the NOD background (25–27). As observed in NOD mice, we found that *Tssp* mRNA was mainly expressed by thymic cDC1 and cDC2, whereas it was barely detectable in splenic cDC1 and cDC2 (Figure 2B, upper row). Thymic pDCs also showed slightly higher levels of *Tssp* mRNA, although this did not reach statistical significance. By contrast, *Ctss* mRNA was expressed by all thymic and splenic DC subsets (Figure 2B, middle row). As observed in NOD mice, the thymic cDC2 subset expressed higher *Ctss* mRNA level than its splenic counterpart. Finally, as observed in NOD mice, *Ctsl* was primarily expressed by thymic and splenic pDCs and to a similar level in both tissues (Figure 2B, lower row). Unexpectedly, the level of *Tssp* transcript was significantly higher in the cDC1 subset in B6 mice as compared with NOD mice; and, conversely, the level of *Ctsl* transcript was significantly higher in the cDC2 subset in NOD mice as compared with B6 mice.

Collectively, these different analyses showed that in the thymus, cDCs express overall higher levels of the antigen-processing enzymes TSSP and CTSS than in the spleen in both autoimmune NOD mice and B6 mice.

### Mature and Immature Thymic Conventional Dendritic Cells Express Similarly High Levels of *Tssp* and *Ctss* mRNA

**Figure 3:**
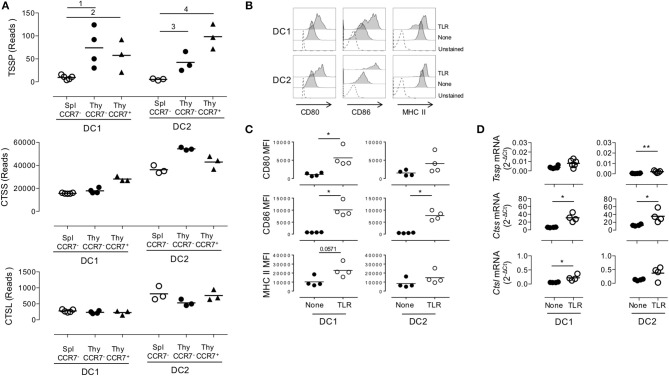
Similar expression levels of *Tssp, Ctss*, and *Ctsl* mRNA in immature and mature conventional dendritic cell (cDCs). **(A)** *Tssp, Ctss*, and *Ctsl* mRNA levels were examined by RNAseq in immature CCR7^−^ thymic (Thy) and splenic (Spl) cDCs and in CCR7^+^ mature thymic cDCs isolated from pools of 10–15 B6 mice. Results are expressed as normalized reads count determined with Deseq2 package. Each symbol corresponds to one RNAseq replicate. Bars indicate significant difference corresponding to a Log2-fold change > 2 and an adjusted *P*-value of (1) 7.1e−7; (2) 1.9e−5; (3) 3e−4; and (4) 1.9e−8. **(B)** Non-obese diabetic (NOD) mice were i.v. injected with lipopolysaccharide (LPS), CpG-B, and poly I:C (toll-like receptor, TLR) or phosphate-buffered saline (PBS) (None); and 20 h later, their spleen cDCs were analyzed by flow cytometry for CD80, CD86, and MHC class II expression. Unstained spleen is shown as control (unstained). **(C)** Comparative median fluorescence intensity for CD80, CD86, and Class II surface expression by control and activated splenic cDC1, cDC2, and pDCs. **(D)** cDC1 and cDC2 were FACS-sorted from spleen of NOD mice i.v. injected with LPS, CpG-B, and poly I:C (TLR) or PBS (None) 20 h earlier. Expression of *Tssp, Ctss*, and *Ctsl* mRNA was assessed by RT-qPCR. Each symbol corresponds to an individual mouse. Significant *P*-values are indicated (*P < 0.05; **P < 0.01).

Given that thymic cDCs are more mature than their splenic cDC counterparts, we considered the possibility that the increased *Tssp* and *Ctss* mRNA levels in thymic cDCs may simply reflect their maturation. To address this issue, we FACS-sorted immature and mature thymic cDCs and immature splenic cDCs on the basis of CCR7 and ESAM expression, and we performed RNAseq analysis (Supplemental Figure 2A and Figure 3). Consistent with the highest expression levels of the co-stimulation markers CD80 and CD86, we found that thymic CCR7^+^/ESAM^+^ cDC1 and cDC2 exhibited a more mature phenotype than splenic and thymic CCR7^−^/ESAM^−^ cDC1 and cDC2 subsets (Supplemental Figures 2B,C). We first made a global analysis of the genes that were differentially expressed by splenic and thymic cDCs (Supplemental Figure 3). As expected, principal component analysis (PCA) of the RNAseq data showed that the cDC1 and cDC2 subsets have distinct transcriptional programs. Although splenic and thymic immature cDC subsets cluster together, mature CCR7^+^ESAM^+^ thymic cDCs were distant. Indeed, using a greater than two-fold change, we identified 4,855 and 3,263 genes that were differentially expressed between immature and mature thymic cDC1 and cDC2, respectively. Nonetheless, splenic and thymic cDCs show some differences, with 877 and 1,173 DEGs between splenic and thymic cDC1 and cDC2, respectively. IPA indicates that these transcriptional signatures correspond to multiple biological pathways (Supplemental Figures 3B–E). We next focused on *Tssp, Ctss*, and *Ctsl*, and we found that overall immature and mature thymic cDC expressed similar levels of *Tssp, Ctss*, and *Ctsl* transcripts (Figure 3A). The only exception was *Tssp* transcripts that were increased in mature thymic cDC2 as compared with immature thymic cDC2, although this did not reach statistical significance. Here again, the level of *Tssp* transcripts was higher in thymic cDCs as compared with splenic cDCs. We did not find, however, in this analysis a significant increase in the number of *Ctss* transcripts in thymic cDC2 as compared with splenic cDC2, although there was a trend (spleen cDC2: 36,205 ± 1,969; CCR7– thymic cDC2: 54,387 ± 708.5; CCR7+ thymic cDC2: 42,909 ± 3,283). Other cathepsins such as Ctsb or Ctsd, in which their role in the class II presentation pathway is less clear, were expressed similarly by immature and mature thymic and splenic cDCs (Supplemental Figure 3F). Similarly, AEP (*Lgmn*) expression was only increased in mature thymic cDCs. Furthermore, the expression of genes coding for class I or class II molecules was very similar between spleen and thymic cDCs and immature and mature thymic cDCs, coherent with the expression of *Ciita* or *Rfx5* in these different populations. cDCs also express several *serpin* encoding cysteine/serine inhibitors, some of which have been shown to inhibit CTSS, CTSL, or papain *in vitro* (28). The expression of some of these serpins is down-regulated in thymic vs. splenic cDCs, suggesting an additional mechanism for increased antigen-presentation by thymic cDCs.

Overall, these analyses show that the overall increased expression of *Tssp* or *Ctss* by thymic cDC as compared with splenic cDC is not simply resulting from the increased maturation of thymic cDC.

We next determined whether, conversely, maturation of splenic cDCs was associated with an increased expression level of *Tssp, Ctss*, and *Ctsl* mRNA. For this experiment, we i.v. injected B6 mice with a combination of LPS, CpG-B ODN1826, and poly I:C; FACS-sorted the cDC1 and cDC2 subsets 20 h later; and analyzed *Tssp, Ctss*, and *Ctsl* mRNA expression by each subset. According to such treatment, cell surface expression of CD80, CD86, and MHC class II was up-regulated, consistent with a successful activation of both splenic cDC1 and in a lower extent cDC2 subsets (Figures 3B,C). We found that *in vivo* TLR stimulation had no effect on the *Tssp* mRNA expression level by splenic cDC1 and induced only a modest increase in its expression in the cDC2 subset that remained, however, significantly lower than that of thymic cDCs (*P* < 0.004; compare Figure 1E and Figure 3D). Similarly, *in vivo* TLR stimulation induced a significant increase in *Ctss* mRNA levels in both splenic cDC1 and cDC2 that reach levels comparable (cDC2) or even higher (cDC1, *P* < 0.012) than their thymic counterparts (compare Figure 1E and Figure 3D). Finally, *in vivo* TLR stimulation increased *Ctsl* mRNA levels in both splenic cDC1 and cDC2 subsets, reaching levels that were significantly higher than those found in thymic cDC1 and cDC2 (*P* < 0.024 and *P* < 0.006, respectively, compare Figure 1E and Figure 3D).

Collectively, these experiments highlight two distinct mechanisms of regulation of *Tssp* and *Ctss* expression by individual cDC subsets. First, the thymic environment was associated with enhanced expression of each protease as compared to the splenic environment, independently of the maturation status of the individual cDC subsets. Second, cDC maturation upon TLR stimulation is mainly associated with an increased expression of *Ctss* mRNA.

### Tissue-Specific Factors Modulate the Expression of Antigen-Processing Enzyme by Dendritic Cell Subsets

**Figure 4:**
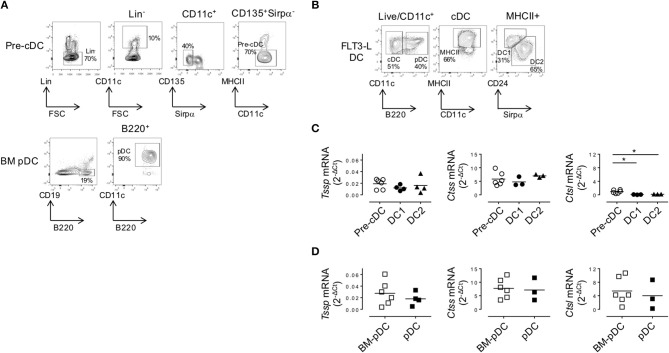
In the bone marrow (BM), precursor of conventional dendritic cells (pre-cDCs) and plasmacytoid DCs (pDCs) express *Tssp, Ctss*, and *Ctsl* mRNA. **(A)** FACS-sorting strategy of pre-cDC (Lin^−^CD11c^+^ class II^−^Sirpα^−^CD135^+^) and pDC (CD19^−^B220^+^CD11c^+^) from BM of non-obese diabetic (NOD) mice. Lin^−^ cells correspond to CD3^−^CD19^−^NKp46^−^Ter119^−^B220^−^ for pre-cDC analysis and CD3^−^CD19^−^NKp46^−^Ter119^−^ for BM pDC analysis. The average percentage of gated cells is shown (*n* = 6). **(B)** NOD BM cells were differentiated *in vitro* in the presence of FLt3L for 9 days; and cDC1, cDC2, and pDCs were gated as indicated. The average percentage of gated cells is shown (*n* = 3). **(C,D)** Pre-cDC and pDC were FACS-sorted from BM or *in vitro*-differentiated cDC1, cDC2, and pDCs. The expression of *Tssp, Ctss*, and *Ctsl* mRNA was analyzed by RT-qPCR on the different sorted populations. The expression levels of *Tssp, Ctss*, and *Ctsl* mRNA in **(C)** BM pre-cDC and *in vitro*-generated cDC1 and cDC2 and **(D)** BM pDC and *in vitro-*generated pDC are shown. Each symbol correspond to a pool of five mice analyzed in five independent experiments. Significant *P*-values are indicated (*P < 0.05).

As discussed above, cDCs derive from a BM precursor, the pre-cDC, that recirculate *via* the bloodstream to lymphoid tissues where the pre-cDCs complete their differentiation into immature cDC1 and cDC2 (6–8). By contrast, pDC differentiation occurs entirely within the BM, and the differentiated pDCs recirculate *via* the bloodstream to seed lymphoid tissues (4). The expression pattern of *Tssp* and *Ctss* mRNA by thymic and splenic cDC suggested that tissue-specific factors might control the expression of these proteases during their final differentiation in the thymus or in the spleen. To further address this issue, we first examined the expression level of *Tssp, Ctss*, and *Ctsl* mRNA by BM pre-cDCs and differentiated BM pDCs. The two BM subsets were FACS-sorted as described in Figure 4A and subsequently subjected to RT-qPCR analysis. We found that both pre-cDCs and BM pDCs expressed relatively high levels of *Tssp* and *Ctss* mRNA (Figures 4C,D). In agreement with their commitment to the cDC lineage, pre-cDCs did not express significant levels of *Ctsl* mRNA, whereas BM pDC did (Figures 4C,D right row). *Tssp* and *Ctsl* expression patterns in pre-cDC and cDC subsets suggested that their expression may be enhanced/sustained by thymic environmental factors, whereas it may be down-regulated by splenic environmental factors. If this hypothesis was correct, one would expect that when generated in the “neutral environment” of *in vitro* cultures of BM precursors in the presence of FLT3L, cDC1, cDC2, and pDCs should maintain a profile similar to that of their respective BM precursors. We therefore cultured BM cells in the presence of FLT3L for 10 days; FACS-sorted the cDC1, cDC2, and pDCs as described in Figure 4B; and subsequently performed RT-qPCR on each subset. We found that *in vitro*-differentiated cDC1 and cDC2 maintained high levels of *Tssp* and *Ctss* mRNA expression and low levels of *Ctsl* mRNA expression (Figures 4C,D). By contrast and similarly to the pDC isolated from the BM, *in vitro*-generated pDCs expressed relatively high levels of the three transcripts (Figures 4C,D, right row).

To ensure that the transcript levels detected in *in vitro*-generated DCs was not due to overt stimulation with FLT3L, we examine *Tssp, Ctss*, and *Ctsl* mRNA levels in the different DC subsets isolated from the spleen and thymus of mice inoculated with B16-Flt3L melanomas. At day 9 post B16-Flt3L injection, the number of thymic and splenic cDCs was increased as was the frequency of the cDC1 subset relative to the cDC2 subset, reflecting enhanced provision of systemic FLT3L (not shown). *Tssp* mRNA expression levels by spleen cDC1 and cDC2 were moderately increased in treated mice as compared with untreated mice (Supplemental Figure 4A, upper row). The expression levels in treated mice remained, however, lower than those detected in the corresponding thymic cDC subset (*P* < 0.004). Similarly, pDCs in the spleen of FLT3L treated mice expressed significantly higher *Tssp* mRNA levels than those from untreated mice, reaching levels found in thymic pDCs. In contrast, FLT3L treatment did not modify the levels of *Ctss* or *Ctsl* mRNA in the different DC subsets, nor did it alter the expression of the different proteases in thymic cDC (Supplemental Figures 2B, 4A,B). Hence, FLT3L signaling has no major effect on the expression of *Tssp, Ctss*, or *Ctsl* mRNA.

**Figure 5:**
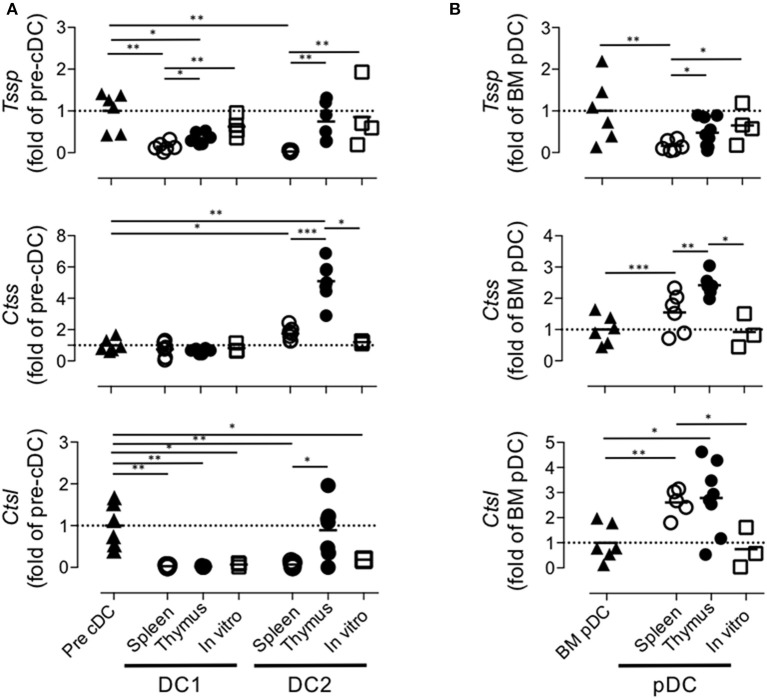
Regulation of *Tssp, Ctss*, and *Ctsl* mRNA expression during dendritic cell (DC) ontogeny. **(A)** Expression of *Tssp, Ctss*, and *Ctsl* mRNA levels in bone marrow (BM) precursor of conventional dendritic cells (pre-cDC), cDC1, and cDC2 isolated from spleen or thymus and *in vitro*-generated cDC are presented as fold expression normalized to pre-cDC. **(B)** Expression of *Tssp, Ctss*, and *Ctsl* mRNA levels in BM pDCs; pDCs isolated from the spleen or thymus; and *in vitro*-generated pDCs are presented as fold expression normalized to BM pDCs. Significant *P*-values are indicated (*P < 0.05, **P < 0.01, ***P < 0.001).

Collectively, these different results indicated that the tissue environment in which pre-cDCs differentiate modulate the level of expression of *Tssp, Ctss*, and *Ctsl* transcripts. To better appreciate this tissue imprinting on the expression of the different proteases, we expressed the mRNA levels of *Tssp, Ctss*, and *Ctsl* detected in the spleen or thymus cDC subsets as fold of its BM precursor (Figure 5A). *Tssp* mRNA levels were reduced following differentiation of pre-cDC into cDC1 or cDC2 but significantly more when differentiation occurs in the spleen than in the thymus (Figure 5A, upper row). *Ctss* mRNA levels were not significantly changed during cDC1 differentiation but were significantly increased during cDC2 differentiation in the spleen and thymus, although this increase was far more important in the thymus as compared with the spleen. Regardless of the tissue considered, *Ctsl* mRNA expression was reduced in differentiated cDCs except for thymic cDC2 that maintain the low pre-cDC level. pDCs, which differentiate in the BM, show a different profile with a marked increase in *Ctsl* transcripts in thymic and splenic pDCs (Figure 5B). However, as observed for cDCs, expression of both *Tssp* and *Ctss* mRNA was higher in thymic pDCs as compared with splenic pDCs.

## Discussion

Thymic cDCs are far more efficient at processing and presenting antigens in the class II pathway than are cDCs from the spleen or lymph node. In this study, we determined whether this may relate to differential expression of proteases known to play a critical role in the generation of peptides for class II presentation. We found that although pre-cDCs express relatively high levels of *Tssp, Ctss*, and *Ctsl* mRNA, their expression was overall down-regulated, as pre-cDCs differentiate in the spleen, whereas it was maintained or enhanced when pre-cDCs differentiate in the thymus. Hence, related to their increased antigen-processing functions, thymic cDCs express high levels of antigen-processing enzymes. Tissue-specific environmental cues, therefore, imprint the expression pattern of a set of antigen-processing enzymes in cDC subsets during their differentiation.

This study also reveals key features of the tissue specification of cDC subsets. First, this extensive analysis corroborates our initial observation of the selective expression of *Tssp* in thymic cDC (20). Interestingly, *Tssp* expression is high in pre-cDCs in the BM, almost extinguished in spleen cDC subsets, and maintained in thymic cDCs or *in vitro*-differentiated pre-cDCs. This difference between spleen and thymic cDCs does not merely reflect a maturation difference, because immature and mature thymic cDCs express comparably high levels of *Tssp* and TLR-dependent maturation of splenic cDCs are not associated with a significant increase in *Tssp* expression. In addition, RNAseq analysis performed by the ImmGen Consortium show that *Tssp*, which is expressed by BM pre-cDC1 and pre-cDC2, is almost extinguished in pre-cDC1 and pre-cDC2 in the spleen. Collectively, these different observations indicate that spleen-specific factors negatively regulate *Tssp* transcription during pre-cDC differentiation. Either these factors are expressed at too low levels in the thymus, or their effect is counteracted by other thymic-specific factors. Interestingly, we found that although the regulation of *Tssp* expression during thymic cDC2 differentiation is conserved between B6 and NOD mice, its expression is significantly higher in the cDC1 subset of B6 mice as compared with NOD mice. The biological significance of this difference is unclear given that TSSP limits the presentation of self-antigen by thymic cDCs and consequently the negative selection of the corresponding CD4 T cell (16–18, 20). Second, we found that *Ctsl* expression is barely detectable in pre-cDCs and further extinguished in the two cDC subset regardless of their tissue origin or whether cDCs are generated in *in vitro* cultures of BM with the exception of thymic cDC2, which maintain the low expression level of their BM precursor. In sharp contrast, *Ctsl* expression is high in BM pDCs and further up-regulated in both thymic and splenic pDCs. These results suggest that *Ctsl* transcription is part of the pDC developmental program. Third, we found that following maturation of splenic cDCs by TLR agonist, the expression of *Ctss* gene is up-regulated to a level comparable with that of thymic cDCs, whereas *Tssp* expression is only modestly increased. This likely reflects differences in the cis-regulatory elements that control expression of these two genes. It is, however, intriguing to see that expression of *Tssp* is confined to the thymus. Indeed, Tssp is expressed at very high levels in cortical thymic epithelial cells and is required for the positive selection of some class II restricted CD4 T cells (20, 29, 30). In addition, Tssp is expressed at low levels by thymic cDCs, and in this thymic stromal subset, TSSP impairs presentation of several self-antigens, thus limiting central tolerance (16–20). Importantly, by limiting central tolerance, TSSP contributes to the diversification of the functional CD4 T cell repertoire specific for foreign antigens (19). However, when expressed in peripheral cDC, TSSP could likewise limit presentation of pathogens-derived antigens, thus restraining the CD4 T cell response and immune responsiveness to pathogens. Given this deleterious effect, it is possible that different regulatory pathways have evolved to control Tssp and Ctss expression.

This study also highlights the lineage-specific program of cDCs. Hence, we found that cDC2 expresses overall higher levels of antigen-processing enzymes than does the cDC1 subset. This is observed for *Tssp* in thymic cDC and for *Ctss* in splenic and thymic cDCs. The cDC1 subset is exquisitely efficient at presenting exogenous antigens in the class I pathway. To optimize cross-presentation, cDC1 has developed several mechanisms to limit endosomal protein degradation. This is achieved at least in part by regulating the phagosome pH through recruitment of v-ATPase and the NADPH oxidase NOX2 at the phagosome membrane (24, 31–33). Here, we show that, in addition, cDC1 expresses lower levels of antigen-processing enzymes.

As a final note, this study provides strong experimental evidence that tissue-specific factors modulate cDC function through the regulation of the expression of antigen-processing enzymes. Finding the extra-cellular and intra-cellular signaling pathways that contribute to this tissue-specific functional specification is critical to develop new DC-based therapies.

## Data Availability Statement

All datasets generated for this study are included in the article/Supplementary Material. All RNA-Seq data are available in NCBI, accession number: GSE144421.

## Ethics Statement

The animal study was reviewed and approved by Midi Pyrénées ethical committee.

## Author Contributions

KM, CH, CM, MG, and SG designed and performed the research and analyzed the data. KM and SG prepared the figures and wrote the manuscript.

### Conflict of Interest

The authors declare that the research was conducted in the absence of any commercial or financial relationships that could be construed as a potential conflict of interest.

## Supplementary Material

Supplemental Figure 1 **(A)** Splenic and thymic cDC were gated as described in Figure 1A. The expression of XCR1 on gated cDC1 and cDC2 is shown. **(B)** Splenic and thymic pDC were gated as described in Figure 1A. The expression of PDCA-1, SiglecH and CCR9 by pDC is shown. Three individual mice were analyzed and the numbers correspond to the mean frequency ± SEM of the cells in the indicated quadrants.

Supplemental Figure 2 **(A)** Flow cytometry strategy used to sort immature and mature cDC. **(B)** Histogram representation and **(C)** Median Fluorescence Intensity quantification of CD80 and CD86 expression by CCR7^+^/ESAM^+^ thymic and CCR7^−^/ESAM^−^ splenic and thymic cDC of B6 mice. Significant *P*-values are indicated (^*^*P* < 0.05, ^**^*P* < 0.01, ^***^*P* < 0.001, ^****^*P* < 0.0001).

Supplemental Figure 3 **(A)** Principal-component analysis (PCA) of RNA-seq expression data from the different conventional dendritic cells populations from the thymus and the spleen of B6 mice. PC1 and PC2 indicate, respectively, principal components 1 and 2. Each point represents individual sample. The top 30 enrichment canonical pathways associated with the Differential Expressed Genes (DGE) between mature and immature thymic cDC1 **(B)** and cDC2 **(C)** and between thymic and spleenic cDC1 **(D)** and cDC2 **(E)** inferred with Ingenuity Pathway Analysis. Horizontal axis indicates the–log (p-value) and vertical axis represents the canonical pathways associated with DEG. The color scale corresponds to the z-score of the canonical pathway. **(F)** Heatmaps showing gene expression levels detected by bulk RNA-seq for the 50 key genes associated with cathepsin, class I and class II presentation pathways. Significant differential expressed genes between immature thymic and splenic cDC or immature and mature thymic cDC are indicated with symbols (±) following the direction of the gene expression variation in the immature thymic cDC or mature thymic cDC column, respectively.

Supplemental Figure 4 FLT3L has no major effect on Tssp, Ctss and Ctsl expression. (**A** and **B**) B16-Flt3l melanomas were injected s.c. into B6 mice. Nine days later, cDC1, cDC2 and pDC were FACS-sorted from spleen **(A)** and total CD11c+ cells were magnetically isolated from thymus **(B)**. Expression of Tssp, Ctss and Ctsl mRNA was examined by RT-qPCR. Each symbol corresponds to one mouse, from 3 independent experiments. Significant *P* values are indicated (^**^P<0.01).
