# Supplementary figures and images for: Tissue-Specific Factors Differentially Regulate the Expression of Antigen-Processing Enzymes During Dendritic Cell Ontogeny
Source: Front Immunol. 2020 Mar 31;11:453. doi: 10.3389/fimmu.2020.00453 (PMC7136460; doi:10.3389/fimmu.2020.00453)

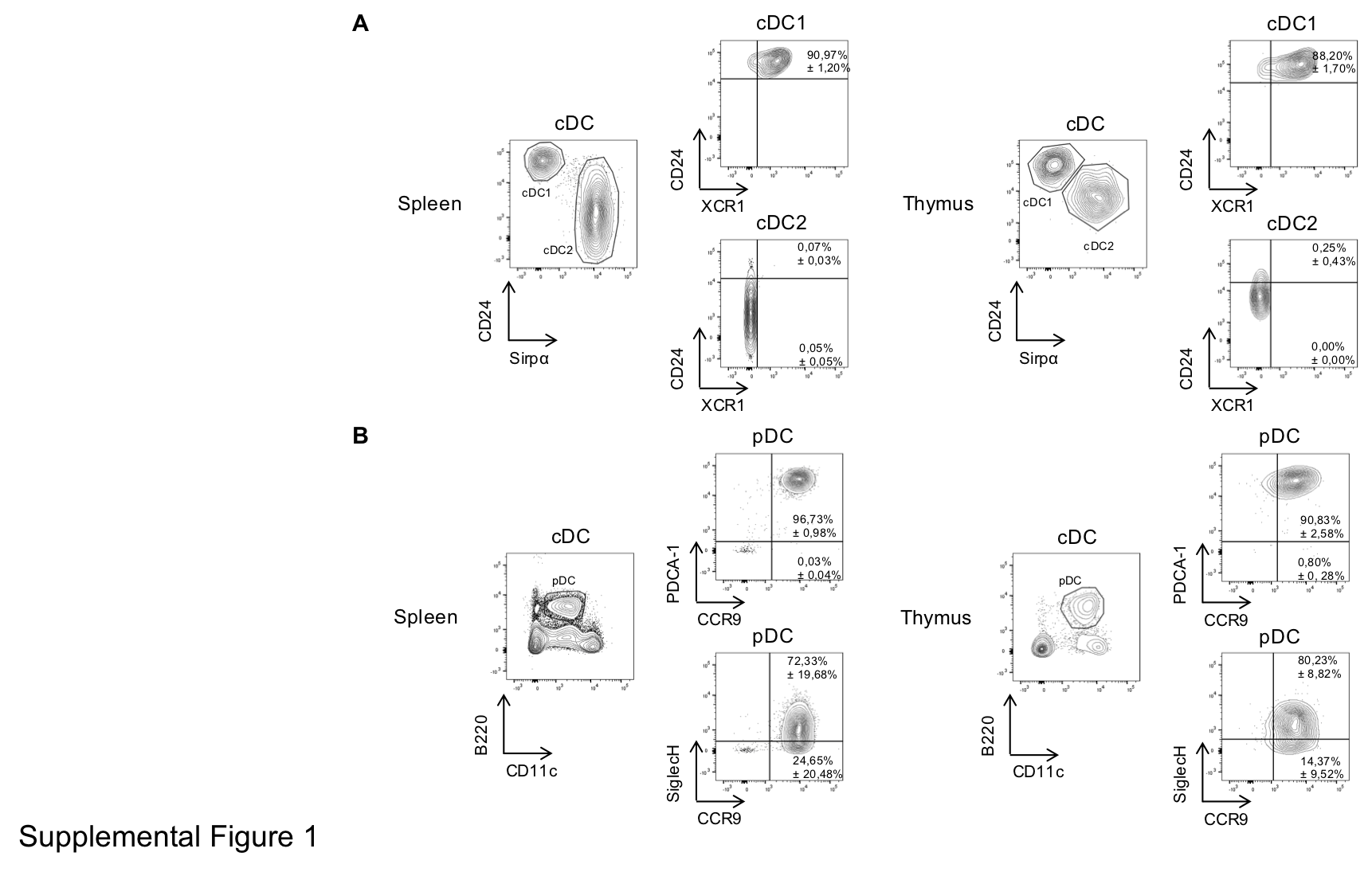

Supplement: Supplemental Figure 1 — (A) Splenic and thymic cDC were gated as described in Figure 1A. The expression of XCR1 on gated cDC1 and cDC2 is shown. (B) Splenic and thymic pDC were gated as described in Figure 1A. The expression of PDCA-1, SiglecH and CCR9 by pDC is shown. Three individual mice were analyzed and the numbers correspond to the mean frequency ± SEM of the cells in the indicated quadrants. [file Image_1.TIFF]

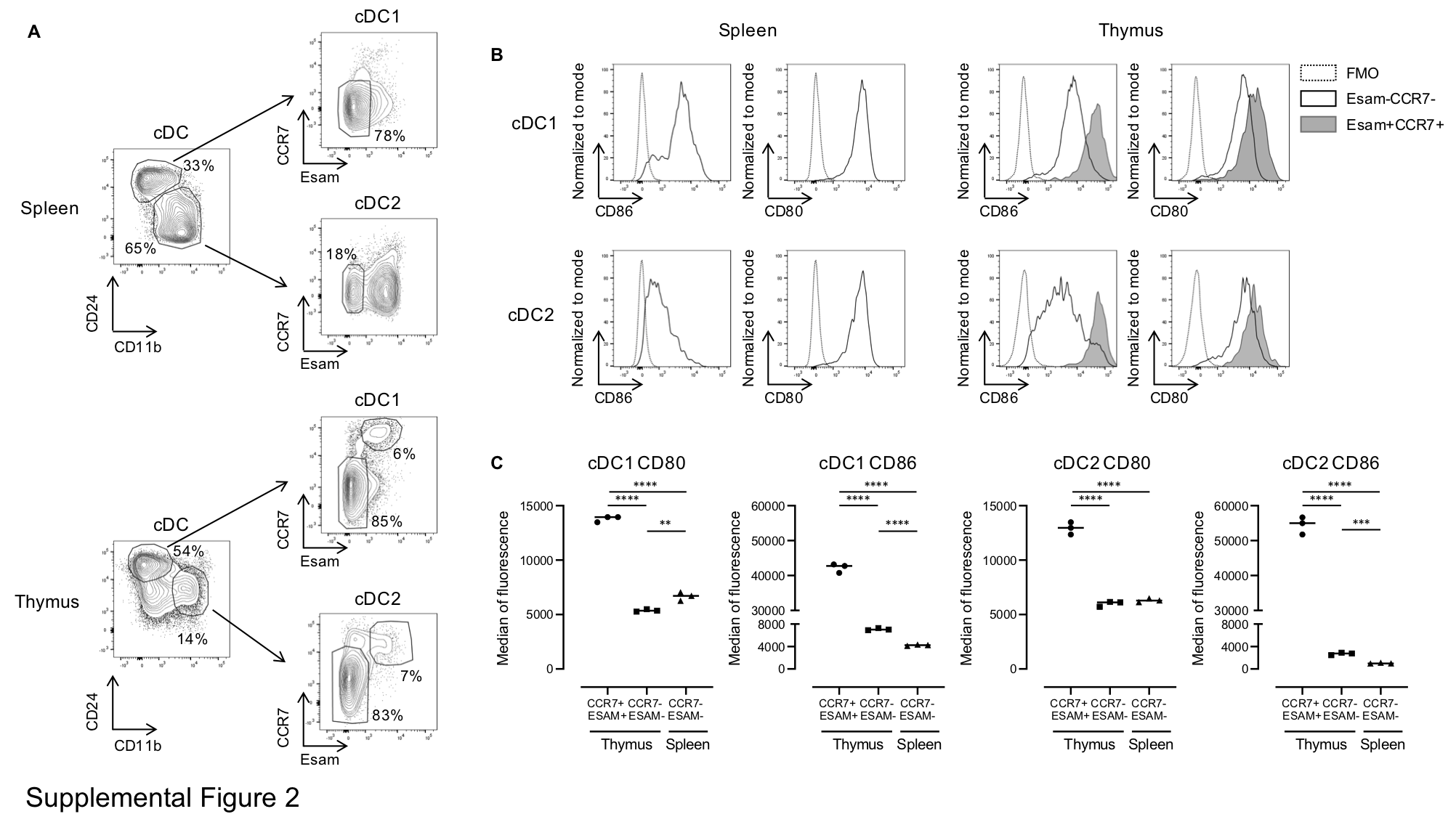

Supplement: Supplemental Figure 2 — (A) Flow cytometry strategy used to sort immature and mature cDC. (B) Histogram representation and (C) Median Fluorescence Intensity quantification of CD80 and CD86 expression by CCR7+/ESAM+ thymic and CCR7−/ESAM− splenic and thymic cDC of B6 mice. Significant P-values are indicated (*P < 0.05, **P < 0.01, ***P < 0.001, ****P < 0.0001). [file Image_2.TIFF]

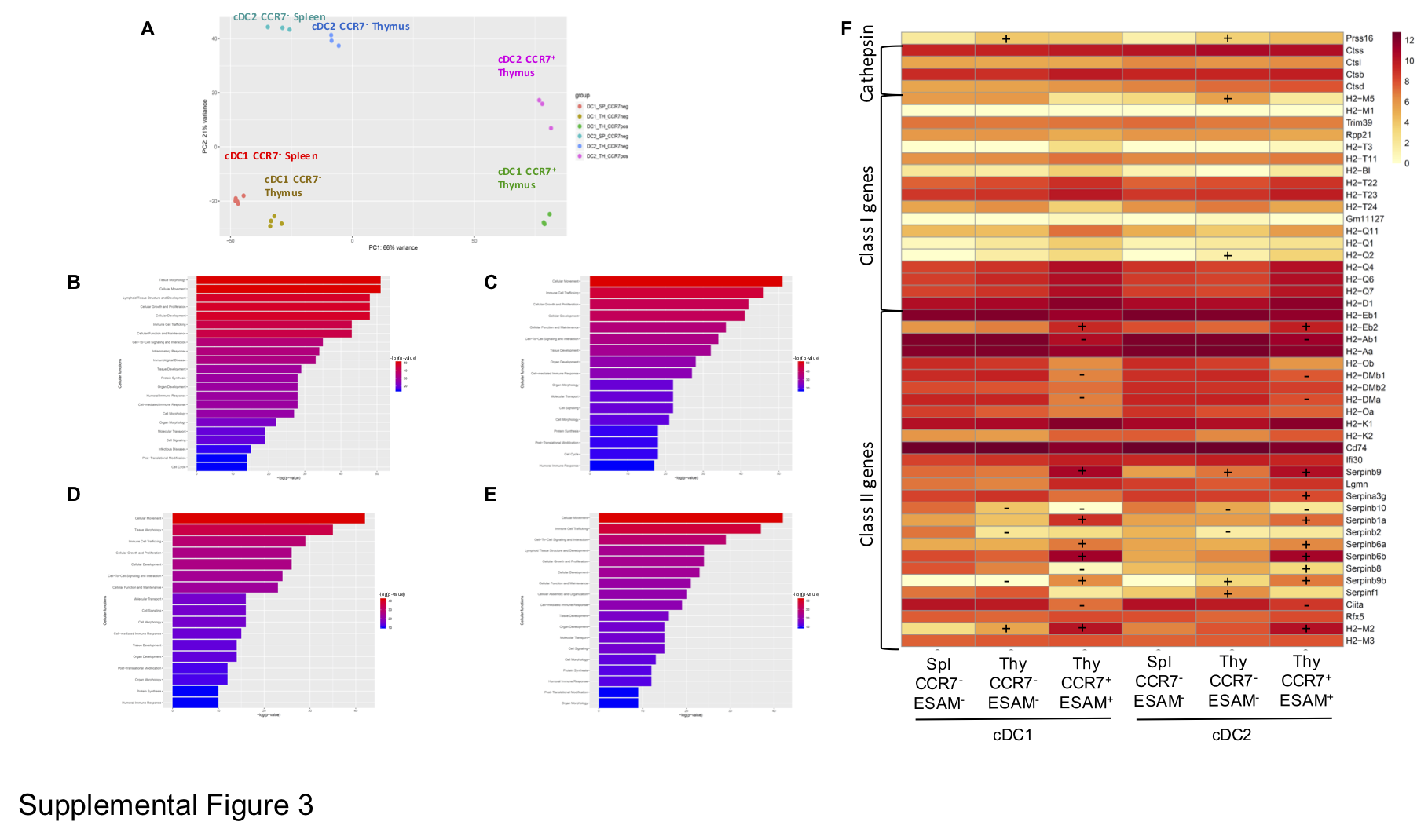

Supplement: Supplemental Figure 3 — (A) Principal-component analysis (PCA) of RNA-seq expression data from the different conventional dendritic cells populations from the thymus and the spleen of B6 mice. PC1 and PC2 indicate, respectively, principal components 1 and 2. Each point represents individual sample. The top 30 enrichment canonical pathways associated with the Differential Expressed Genes (DGE) between mature and immature thymic cDC1 (B) and cDC2 (C) and between thymic and spleenic cDC1 (D) and cDC2 (E) inferred with Ingenuity Pathway Analysis. Horizontal axis indicates the–log (p-value) and vertical axis represents the canonical pathways associated with DEG. The color scale corresponds to the z-score of the canonical pathway. (F) Heatmaps showing gene expression levels detected by bulk RNA-seq for the 50 key genes associated with cathepsin, class I and class II presentation pathways. Significant differential expressed genes between immature thymic and splenic cDC or immature and mature thymic cDC are indicated with symbols (±) following the direction of the gene expression variation in the immature thymic cDC or mature thymic cDC column, respectively. [file Image_3.TIFF]

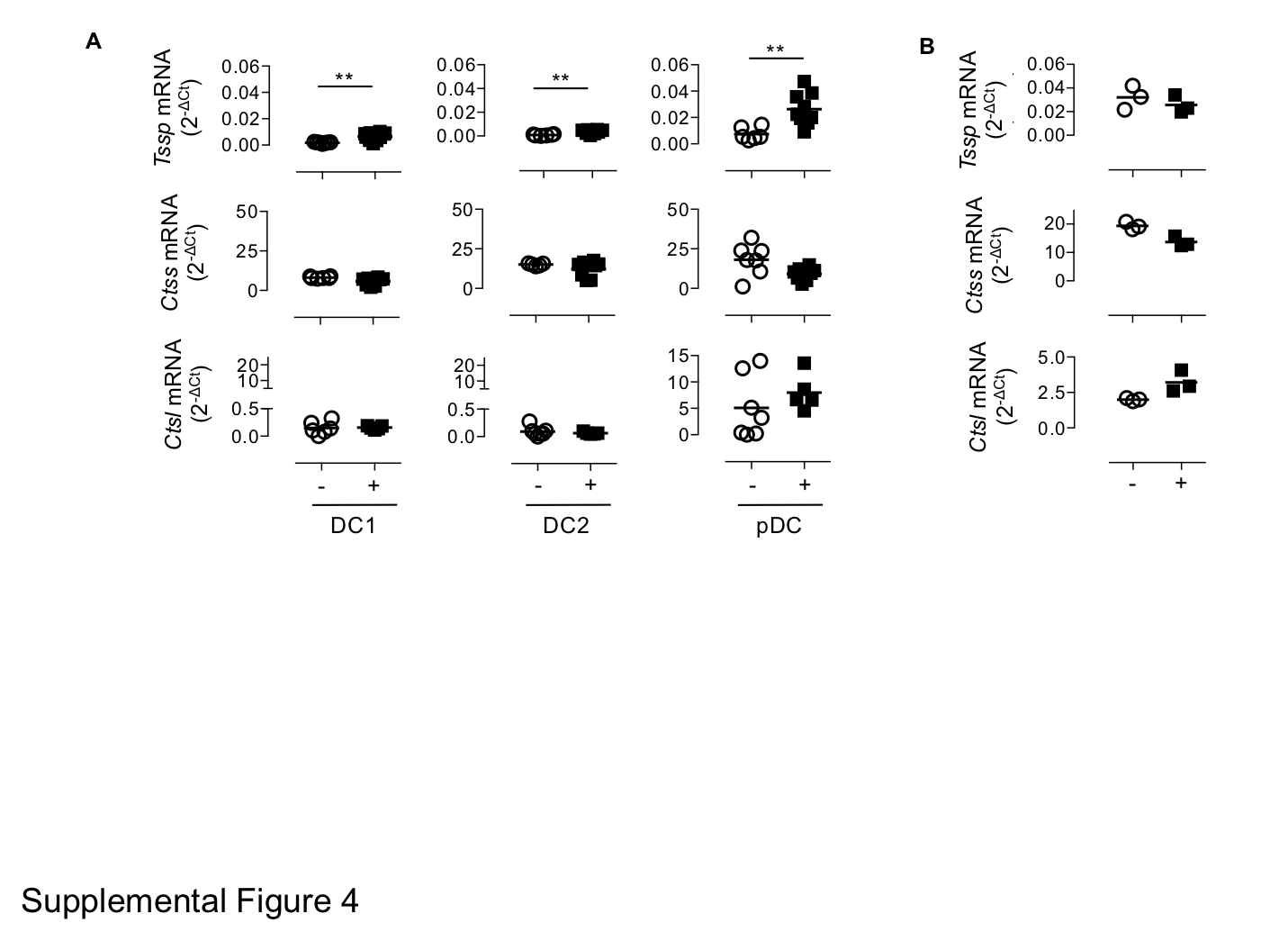

Supplement: Supplemental Figure 4 — FLT3L has no major effect on Tssp, Ctss and Ctsl expression. (A and B) B16-Flt3l melanomas were injected s.c. into B6 mice. Nine days later, cDC1, cDC2 and pDC were FACS-sorted from spleen (A) and total CD11c+ cells were magnetically isolated from thymus (B). Expression of Tssp, Ctss and Ctsl mRNA was examined by RT-qPCR. Each symbol corresponds to one mouse, from 3 independent experiments. Significant P values are indicated (**P<0.01). [file Image_4.TIFF]
